# Cytometric detection of antigen-specific IFN-γ/IL-2 secreting cells in the diagnosis of tuberculosis

**DOI:** 10.1186/1471-2334-9-99

**Published:** 2009-06-23

**Authors:** Valeria Sargentini, Sabrina Mariotti, Stefania Carrara, Maria Cristina Gagliardi, Raffaela Teloni, Delia Goletti, Roberto Nisini

**Affiliations:** 1 Dipartimento di Malattie Infettive, Parassitarie e Immunomediate, Istituto Superiore di Sanità, 00161 Roma, Italy; 2 Translational Research Unit, Department of Experimental Research, Istituto Nazionale Malattie Infettive Lazzaro Spallanzani- IRCCS Rome, 00149 Roma, Italy

## Abstract

**Background:**

The purpose of this study was to further characterize the immune response to *Mycobacterium tuberculosis *(Mtb) antigens, in order to provide new insight into host-pathogen interactions in tuberculosis (TB), and to offer tools for a more accurate diagnosis of the different stages of TB.

**Methods:**

T-cell responses to Bacillus Calmette-Guérin (BCG), purified protein derivative (PPD), early secretory antigenic target-6 (ESAT-6) protein and culture filtrate protein-10 kDa (CFP-10) were measured in terms of interferon (IFN)-γ and interleukin (IL)-2 release, using a novel flow cytometric cell-secreting cytokine detection technique. The study was conducted on peripheral blood mononuclear cells (PBMC) obtained from active TB patients, latently TB infected individuals, and healthy donors. IL-10 and IL-17 were also measured to test their possible role as indicators of disease activity.

**Results:**

We confirmed that the enumeration of IFN-γ releasing cells upon Mtb-specific stimulation is sufficient to identify TB patients and that CD8+ T cells concur to IFN-γ secretion. IL-2 secreting cells were more frequently observed in latent TB infected individuals compared to active TB patients, suggesting that measurement of cells secreting this cytokine could be a marker of disease stage. No discriminating role was associated to IL-10 and IL-17 release in TB patients.

**Conclusion:**

Our data indicate that the flow cytometric cytokine-secreting cell detection technique may be envisaged as an additional tool for TB diagnosis allowing the analysis of the immune response to *M. tuberculosis*-related antigens in the different stages of TB.

## Background

Tuberculosis (TB) is one of the most prevalent bacterial infections of humans worldwide [1], but the large majority of *M. tuberculosis *infected individuals are asymptomatic. In fact, healthy individuals who get infected by *M. tuberculosis *for the first time usually develop a mild disease: primary TB. These individuals clinically heal, but are unable to clear the bacterium that remains dormant within granulomas or cellular reservoirs [2-4]. This latent TB infection may have a lifelong persistence, without clinical relevance [5]. In some subjects, due to concomitant infections, malnutrition or other factors, latent *M. tuberculosis *may reactivate causing a post-primary TB that usually develops chronically, unless treated with antibiotics [6,7].

One of the main problems in the fight against TB is the correct diagnosis of its different stages. Tuberculin skin test (TST) is the most used screening method for the diagnosis of *M. tuberculosis *infection, even though a prior vaccination with Bacillus Calmette Guérin (BCG) can lead to false positive results using this test. Other widely recognized limits of TST are the false negative tests due to anergy or immune deficiency and its inability to distinguish latently infected individuals from patients with active TB. Moreover, the site of the skin test must be examined by a health professional within 48 – 72 hours to determine the result of the test [8,9]. Newer assays based on the release of interferon (IFN)-γ upon specific antigen stimulation (IGRAs) have recently become available [10-12], but their enhanced sensitivity and specificity compared to TST [8,13] have not been demonstrated yet. These tests take advantage by the use of early secretory antigenic target-6 (ESAT-6) and culture filtrate protein-10 kDa (CFP-10), proteins that are encoded within the region of difference 1 (RD1) of *M. tuberculosis *but not BCG genome, and thus are not synthesized by BCG substrains or most environmental mycobacteria, with few exceptions [14-16]. In addition, some of these tests are commercially available and approved for TB infection diagnosis. However, IGRAs can discriminate vaccinated from *M. tuberculosis*-infected individuals, but not patients affected by active TB disease from individuals with latent TB infection. Lastly, the instrumentation required to perform some of IGRAs is not available in all laboratories [17,18].

A possible contribution to discriminate the different TB stages could be envisaged in a more accurate definition of the immunological response to selected *M. tuberculosis *antigens. In fact, the immune response should differ according to the antigen load, i.e. to active disease or latent infection. Since effector T cells (TEM) have a limited lifespan, it is possible to hypothesize that in the presence of active bacterial replication, i.e. active disease, their frequency should be increased in comparison to central memory T cells (CEM) in peripheral blood mononuclear cells (PBMC), whereas CEM should be predominant in latent infections. Beside phenotypic surface markers, memory cells can be distinguished according to their differential cytokine secretion: TEM secrete IFN-γ alone while CEM interleukin (IL)-2 or IFN-γ and IL-2. Moreover, they show different kinetics of stimulation: rapid (hours) for TEM and longer (days) for CEM [19,20]. In this line, it has been recently shown that *M. tuberculosis *specific T cell response in patients with a non-replicating *M. tuberculosis *infection is mainly characterized by IL-2-secreting T cells [21].

In this study we investigate the possible contribution of active cytokine-secreting memory T cell enumeration as a diagnostic tool for TB infection and disease using a modified commercially available method [22,23].

## Methods

### Study population

Patients admitted to the infectious diseases ward at the National Institute for Infectious Diseases 'L. Spallanzani', Rome, Italy, between 2007 and 2008 were evaluated for enrolment. Individuals underwent TST administered by the Mantoux procedure using 5 IU of purified protein derivative (PPD) (Chiron, Siena, Italy) and after 72 h an induration of at least 10 mm was scored positive. TST and *in vitro *T cell assays were performed at the same time within a week from the admission. Enrolled individuals were classified as 'active TB' when the diagnosis was confirmed by a positive *M. tuberculosis *culture from sputum specimens. For those with a diagnosis of extra-pulmonary TB, a polymerase chain reaction was positive for *M. tuberculosis *on biopsies or on pleural fluid. None of the patients resulted positive for human immunodeficiency virus (HIV) screening. The "healthy subjects" were laboratory personnel from Italy with no known exposure to *M. tuberculosis*.

Latent TB infection was defined by a positive response to TST and to the QuantiFERON TB Gold in tube (Cellestis Ltd, Victoria, Australia) in exposed individuals without signs of active disease. The study was approved by the Ethical Committee of the Institute and all enrolled individuals gave their written informed consent. Demographic and clinical characteristics of enrolled individuals are reported in table 1.

**Table 1:** Demographic and clinical characteristics of enrolled individuals

	Active TB N. 20	LTBI subjects N. 11	Healthy subjects N. 12	Total N. 43
**Female (%)**	10 (50)	4 (36.4)	7 (58.3)	21 (48.8)
**Age, median (years)**	35.7	42.0	45.1	40.9
**Ethnicity:**				
***West Europe *(%)**	10 (50)	9 (81.8)	12 (100)	31 (72)
**East Europe (%)**	4 (20)	-	-	4 (9.3)
**South America (%)**	1 (5)	1 (9)	-	2 (4.6)
**Africa (%)**	3 (15)	-	-	3 (6.9)
**Asia (%)**	2 (10)	1 (9)	-	3 (6.9)
**BCG vaccinated (%)**	7 (35)	0 (0)	1 (8.3)	8 (18.6)
**TST positive (%)**	12 (60)	11 (100)	1 (8.3)	24 (55.8)
**TB localization:**				
***Pulmonary (%)***	17 (85)	-	-	17 (39.5)
**Extra-pulmonary (%)**	3 (15)	-	-	3 (6.9)
**Positive by QuantiFERON-TB Gold in tube (%)**	15 (75)	11 (100)	0 (0)	26 (60.5)

**LTBI**: Latent TB infection

### Growth of mycobacteria

BCG (ATCC 27291) was grown in Middlebrook 7H10 agar (Difco Laboratories, Detroit, MI) at 37°C under a humidified 5% CO_2 _atmosphere for two weeks and colony-forming units were counted as previously described [24].

### Cell isolation and culture

PBMC were purified from heparinized blood obtained on a density gradient (Lymphoprep, Nicomed Pharma AS, Oslo, Norway) as previously described [25]. PBMC were resuspended in RPMI 1640, supplemented with 100 U/ml kanamycin, 1 mM glutamine, 1 mM sodium pyruvate, 1% nonessential amino acids, and 5% human AB serum (complete medium: CM) (all from Euroclone Ltd., Wetherby Yorkshire, UK), and cultured at 5 × 10^5 ^cells/ml for 18 and 72 hours. Cell cultures were treated with staphylococcal enterotoxin A (SEA, Sigma Chemical Co. St. Louis, USA) at 0.5 μg/ml, PPD (Statens Serum Institut, Copenhagen, DK) at 5 μg/ml, a pool of ESAT-6 protein (Statens Serum Institut) and CFP-10 (Lionex, Braunschweig, Germany) at 5 μg/ml and 2 μg/ml respectively, and BCG at the multiplicity of infection 1:1 [26]. Negative controls included un-stimulated PBMC.

Cell-culture supernatants were collected for cytokine determination by ELISA. 

#### QuantiFERON TB Gold

QuantiFERON TB Gold in tube assay was performed as indicated by the manufacturer.

### Enumeration of cytokine-secreting-cells by flow cytometric analysis

#### Secretion Assay

Antigen specific T cells were analyzed using IFN-γ, IL-2 and IL-10 Secretion Assays (Miltenyi Biotec, Bergisch Gladbach, Germany). After 18 and 72 hours of culture, cells were incubated for 5 minutes on ice with IFN-γ and IL-2 or IL-10 Catch Reagents (Miltenyi, amounts according to manufacturer's instructions). Cells (10^5 ^cells/ml) were then diluted adding warm (37°C) CM, and incubated for 45 minutes under slow continuous rotation at 37°C and 5% CO_2_. Cells were further incubated for 10 minutes with the Detection Antibodies anti-IFN-γ mAb (mouse IgG1) FITC-conjugated, anti IL-2 mAb (mouse IgG1) PE-conjugated, or anti   IL-10 mAb (mouse IgG1) APC-conjugated (Miltenyi, amounts according to manufacturer's instructions). In addition, cells were incubated with a mAb anti-CD4 (PE-Cy7 conjugated, clone SK3, mouse IgG1), anti-CD8 (APC-Cy7 conjugated, clone SK1, mouse IgG1), anti-CD16 (PE-Cy5, clone 3G8, mouse IgG1). All the mAbs and isotype-matched controls were purchased from BD PharMingen, San Diego, CA.

Flow cytometry was performed on a FACSCanto flow cytometer (Becton Dickinson, San Jose, CA) analyzing 50000 viable cells and data were analyzed with the BDFACS Diva software. The percentages of IFN-γ^+^, IL-2^+^, IFN-γ^+^/IL-2^+ ^(double positive) or IL-10^+ ^events were determined with quadrant statistics on CD16^- ^population or on CD4^+ ^and CD8^+ ^gated cell population. The characteristics of the method used do not allow a linear quantification of cytokine secreting cells when their frequency is higher than 5%. Since our aim was not to determine the absolute number of cytokine secreting cells but to analyze the antigen dependence of their synthesis, we assigned the arbitrary value of 6% to all the frequencies of cytokine secreting cells higher than 5%. This correction does not interfere with the antigen-dependence of results and does not influence the interpretation of data for statistical analysis and representation of results for diagnostic purposes.

#### Intracellular staining

PBMC were incubated with brefeldin A (Golgi plug) (Pharmingen, San Diego, CA, USA) added during the last 3 hours of culture. Cells were then stained with PE conjugated mAb anti-CD4 (clone SK3, mouse IgG1) or anti-CD8 (clone SK1, mouse IgG1) from BD Pharmingen and were incubated with FITC-conjugated anti-IFN-γ (clone 4S.B3, mouse IgG1) from BD Pharmingen after treatment with Cytofix-Cytoperm reagent (BD Pharmingen) according to the manufacturer's instructions.

Flow cytometry was performed on a FACSCanto flow cytometer (Becton Dickinson, San Jose, CA) analyzing 50000 viable cells and data were analyzed with the BDFACS Diva software.

### IL-17 and IL-10 detection

IL-17 and IL-10 were detected by commercial Enzyme Linked ImmunoSorbent Assay (ELISA) kits for human IL-17 and IL-10 according to the manufacturer's instructions (R&D Systems, Minneapolis, MN, USA). Detection limit of the assays is 15 pg/ml.

### Statistical Analysis

Control group included 12 healthy subjects. Comparison of frequencies of IFN-γ, IFN-γ/IL-2 and IL-2-secreting cells and ELISA measures among groups were tested using the Mann-Whitney *U *test. A *p *< 0.05 was considered significant.

Receiver operating characteristic (ROC) curves were constructed plotting sensitivity versus 100-specificity evaluated at several different diagnostic thresholds. The area under the curve (AUC), the corresponding 95% confidence interval (CI) and *p*-value were calculated. Optimum cut-off values, with the combination of the highest sensitivity and specificity, were calculated.

All the analyses were performed using GraphPad Prism Software 4 (San Diego, CA).

## Results

### Analysis of *M. tuberculosis*-specific cytokine-secreting T lymphocytes

**Figure 1:**
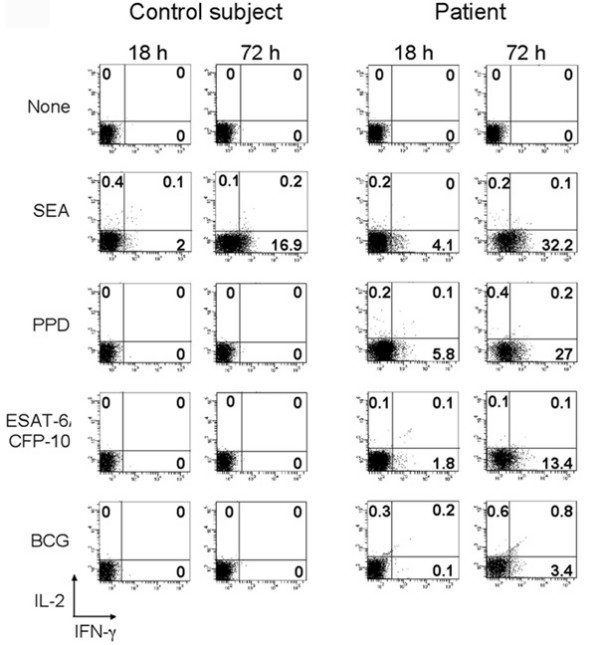
**Antigen specific cytokine secretion in PBMC of active TB patients**. PBMC from control subjects and active TB patients were cultured for 18 and 72 hours after stimulation with SEA, PPD, ESAT-6/CFP-10 and BCG or left un-stimulated (none). Cells were then treated as specified in the M&M section for analysis of secreted cytokines (IFN-γ and IL-2) and evaluated on CD16^- ^gated cells by flow cytometry. Data from one representative experiment are reported. Numbers indicate the percentages of positive cells in the relative quadrants.

**Figure 2:**
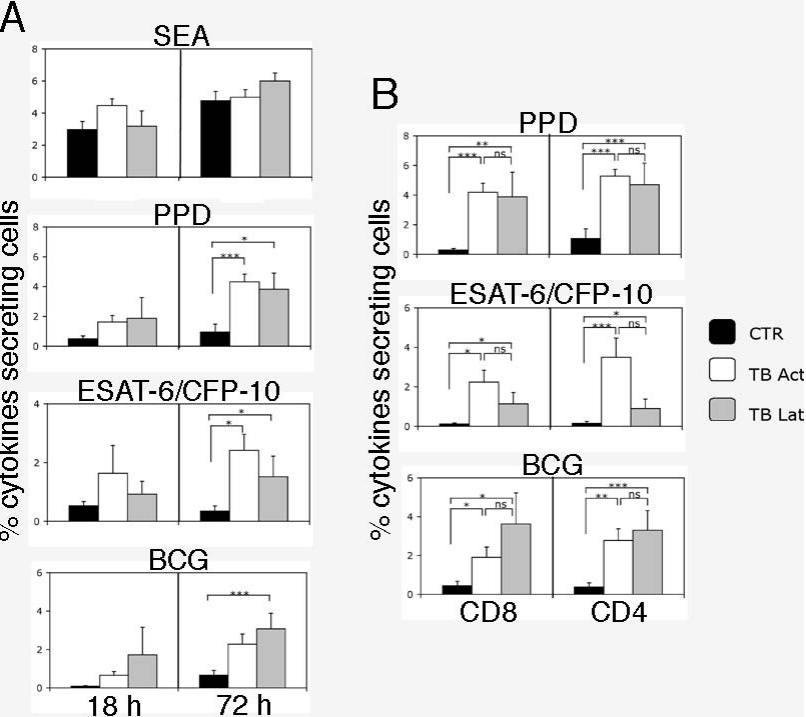
**Analysis of antigen-specific cytokines secreted by T cells**. A) Frequency of cytokine secreting cells after 18 (left panel) or 72 (right panel) hours of culture with the positive control SEA, PPD, ESAT-6/CFP-10 or BCG in PBMC of controls (CTR, black bars), patients with active TB (TB Act, white bars) or individuals with latent TB infection (TB Lat, grey bars). Columns represent median values ± standard error of the frequency (%) of the total cytokines (IFN-γ, IL-2 and IFN-γ/IL-2) secreting cells calculated on CD16^- ^total T cells. Only the statistically significant (*p*-values < 0.05) differences among the indicated populations were reported. * indicates *p *< 0.05; *** indicates *p *< 0.005. B) Frequency of cytokine secreting cells among the CD8^+ ^(left panel) or the CD4^+ ^(right panel) cells in PBMC of controls (CTR), patients with active TB (TB Act) or individuals with latent TB infection (TB Lat) after 72 hours of culture with PPD, ESAT-6/CFP-10 or BCG. Columns represent median values ± standard error. Differences among the indicated populations were considered significant when the *p*-values were < 0.05. * indicates *p *< 0.05; ** indicates *p *< 0.01; *** indicates *p *< 0.005; n.s. = not significant.

PBMC from patients and control individuals were stimulated with SEA, PPD, ESAT-6/CFP-10 and BCG or left un-stimulated for 18 or 72 hours and then analysis of cytokine secreting cells was performed. As shown in one representative example reporting the frequency of IFN-γ and IL-2-secreting cells (Figure 1) and detailed in figure 2A, after 18 hours of culture a noticeable but not statistically significant difference in the number of cytokine secreting T cells was detectable between groups in cultures stimulated with *M. tuberculosis *antigens or BCG, but not in those stimulated with SEA or left un-stimulated. Due to the reduced time of *in vitro *stimulation, it is reasonable to believe that responder cells were TEM. The frequency of cytokine secreting T cells increased when analysis was performed after 72 hours of culture (Figure 1 and 2A). The statistical analysis of the results indicates that this method allows the discrimination of active TB and latent TB infection from control individuals but not between active TB patients and subjects with latent TB infection upon *M. tuberculosis*-specific stimulation. Due to the best performance of the test obtained after 72 hours, we decided to focus our attention on this time point for the rest of the study.

Gating the population on CD4^+ ^and CD8^+ ^T cells gave the opportunity to analyze their contribution to the global cytokine secretion after *M. tuberculosis*-specific stimulation. The analysis of cytokine secretion by single T cell subpopulations mirrored the analysis on the whole T cells (Figure 2A) and antigen-specific cytokine secreting T lymphocytes were present not only in the CD4^+ ^but interestingly also in the CD8^+ ^subpopulation (Figure 2B). Although both CD4^+ ^and CD8^+ ^cytokine secreting cells were also detectable using the conventional intracellular cytokine detection method (data not shown), their frequency was higher using our method.

### Analysis of IFN-γ secretion by *M. tuberculosis*-specific T lymphocytes

**Figure 3:**
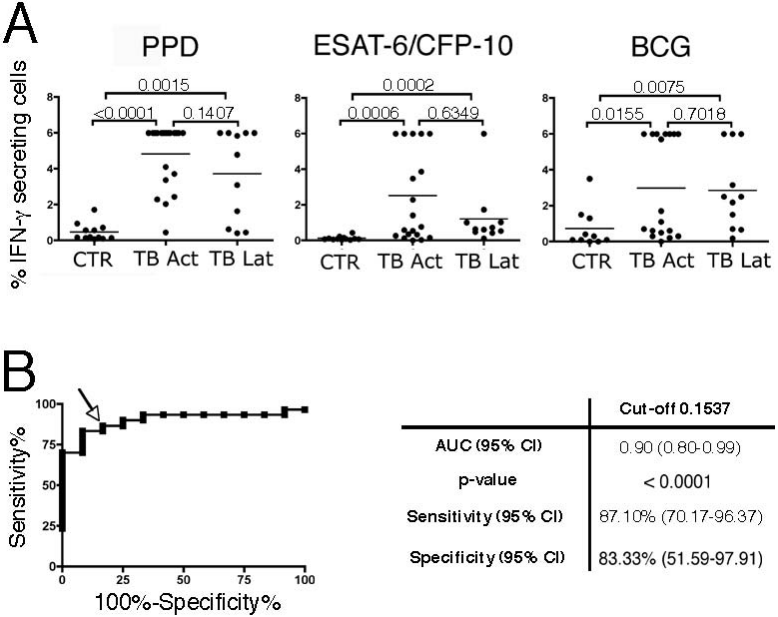
**Identification of *M. tuberculosis*-infected individuals according to IFN-γ secretion**. A) Distribution of IFN-γ secreting T cells in PBMC of controls (CTR), patients with active TB (TB Act) or individuals with latent TB infection (TB Lat) stimulated for 72 hours with PPD, ESAT-6/CFP-10 or BCG. Horizontal lines indicate the median. Differences between the indicated populations were considered significant when the *p*-values (indicated by the numbers) were < 0.05. B) ROC curve (on the left) was generated plotting sensitivity (%) versus 100%-specificity% to examine the effect of different cut-points of the percentage of ESAT-6/CFP-10-specific IFN-γ secreting T cells in TB patients, irrespective of disease stage, versus control subjects. Squares indicate the various percentage cut-off values used to calculate the corresponding sensitivity and specificity. Arrow indicates the optimum cut-off (0.1537%) with the best combination of sensitivity and specificity. On the right the AUC and the statistical significance of the ROC curve and the sensitivity and specificity corresponding to the optimum cut-off are indicated.

Figure 3A reports the distribution of IFN-γ secreting T cells in control subjects and patients with active TB or latent TB infection upon specific stimulation. Statistic analysis of the results indicates that this method allows the discrimination of active TB and latent TB infection from control individuals with all the stimuli used. However, the test did not allow the discrimination between active TB patients and subjects with latent TB infection.

ROC curves are used in diagnostic accuracy studies to depict the pattern of sensitivities and specificities observed when performance of the test is evaluated at several different diagnostic thresholds and to ascertain the optimum cut-off with the best combination of sensitivity and specificity. In order to analyze the performance of our test, a ROC curve was constructed with different cut-offs of percentage of IFN-γ secreting cells upon stimulation with ESAT-6/CFP-10 choosing *M. tuberculosis*-infected (active TB and latent TB infection) and healthy individuals as comparator groups. The curve was positioned near the desirable upper left corner of the ROC space indicating a good performance (*p*-value < 0.0001) of IFN-γ secreting cell enumeration after ESAT-6/CFP-10 stimulation with our test in the discrimination of individuals with TB infection (Figure 3B).

The optimum cut-off (0.1537%) was chosen to compare the diagnostic value of IFN-γ secretion analyzed at single cell level upon stimulation with ESAT-6/CFP-10 with the standard QuantiFERON (cut-off = 0.35 U.I./ml). Our test identified 27 out of 31 *M. tuberculosis*-infected individuals (87.1%), while QuantiFERON TB Gold in tube identified 26 out 31 *M. tuberculosis*-infected individuals (83.87%).

### Analysis of IL-2 secretion by *M. tuberculosis *specific T lymphocytes

The concomitant detection of IL-2 and IFN-γ on *M. tuberculosis*-specific T cells allowed the identification of three different populations: cells secreting only IFN-γ or IL-2, respectively, and cells secreting both cytokines.

**Figure 4:**
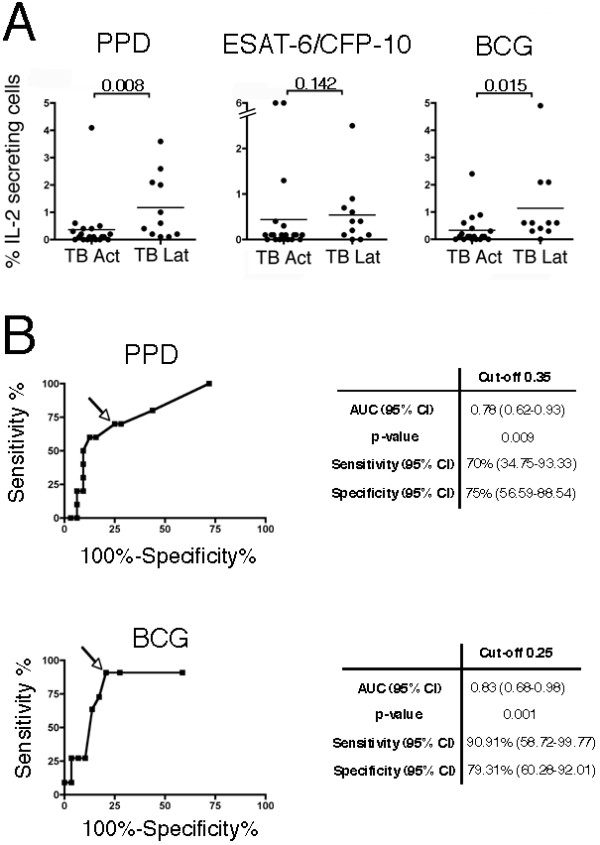
**Identification of latently *M. tuberculosis*-infected individuals according to IL-2 secretion upon stimulation with PPD and BCG**. A) Distribution of the frequencies (%) of IL-2-secreting T cells in PBMC of patients with active TB (TB Act) or individuals with latent TB infection (TB Lat) stimulated for 72 hours with PPD, ESAT-6/CFP-10 or BCG. Horizontal lines indicate the median. Differences among the populations were considered significant when the *p*-values (indicated by the numbers) were < 0.05. B) ROC curves (on the left) were generated plotting sensitivity (%) versus 100%-specificity% to examine the effect of different cut-points of the percentage of PPD-specific (on the top) or BCG-specific (on the bottom) IL-2 secreting T cells in latently infected individuals versus both control subjects and active TB patients. Squares indicate the various percentage cut-off values used to calculate the corresponding sensitivity and specificity. Arrows indicate the optimum cut-offs (0.35% and 0.25% for PPD and BCG, respectively) with the best combination of sensitivity and specificity. On the right the corresponding AUC and statistical significances of the ROC curves and the sensitivity and specificity corresponding to the optimum cut-offs are indicated.

In order to evaluate the diagnostic value of IL-2-secreting cell enumeration, we analyzed the distribution of T cells, that actively secrete IL-2 irrespective of their IFN-γ secretion, among groups. Interestingly, the percentage of IL-2-secreting T cells in individuals with latent TB was statistically higher than in patients with active disease when PPD (*p *= 0.008) or BCG (*p *= 0.015) were used as stimuli (Figure 4A).

To validate the possibility to discriminate individuals with latent TB infection from both controls and patients with active TB using PPD or BCG-induced IL-2 secretion, we constructed the corresponding ROC curves. IL-2-secreting cell enumeration after PPD or BCG stimulation with our test was associated with a good performance in the discrimination of individuals with latent TB infection from both active TB patients and control subjects (Figure 4B).

### Analysis of IL-17 and IL-10 secretion following stimulation with *M. tuberculosis *antigens

**Figure 5:**
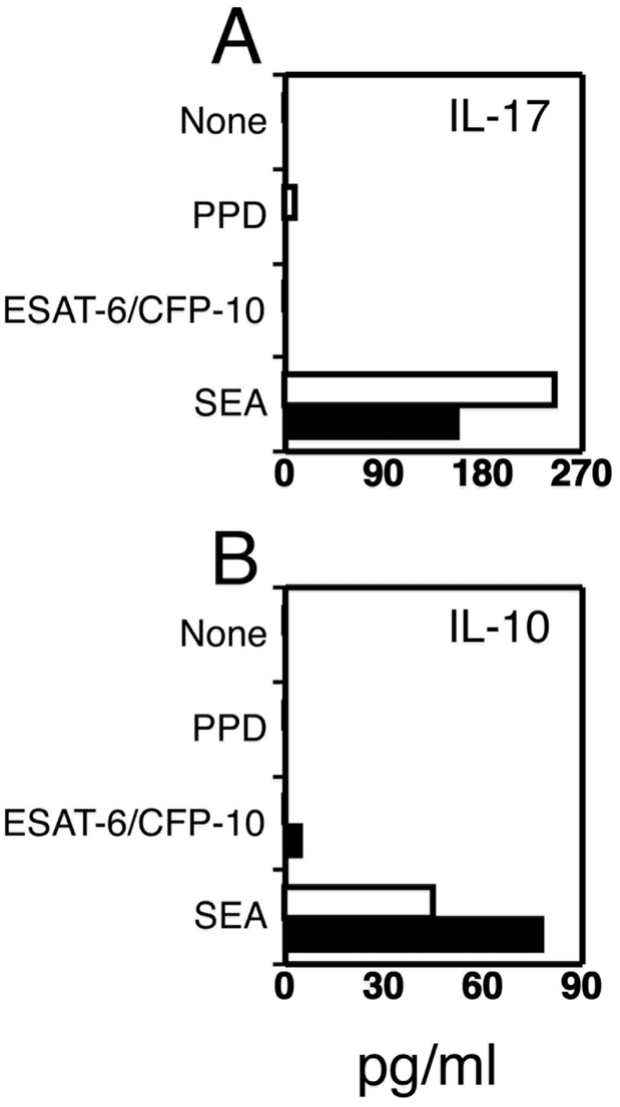
**Secretion of IL-17 and IL-10 by PBMC stimulated with *M. tuberculosis *antigens**. PBMC of controls (black bars, n = 6) or patients with TB (white bars, n = 14) were activated with SEA, PPD or ESAT-6/CFP-10 or left un-stimulated (None). After 72 hours supernatants were collected and examined for A) IL-17 and B) IL-10 content by ELISA. Detection limit of the assays is 15 pg/ml. Histograms represent the median value of cytokine secretion after each stimulus expressed in pg/ml.

To test the possible contribution of IL-17 secretion in the diagnosis of the different forms of TB, we measured the content of this cytokine in supernatants of 72 hour cultures of stimulated PBMC from 14 active TB patients and 6 healthy controls. The difference in the secretion of IL-17 with or without stimulation with SEA (positive control) was statistically significant (*p *< 0.01) but differences in the secretion of IL-17 between controls and TB patients were not significant (Figure 5A). Also, no significant difference was observed between controls and active TB patients stimulated with PPD and ESAT-6/CFP-10 (Figure 5A). PPD-induced IL-17 secretion was higher than in healthy controls in three (average of 104 pg/ml) out of 14 patients, but we could not identify any clinical characteristic of these subjects correlating with the increased IL-17 release.

We also evaluated the frequency of IL-10 producing cells by flow cytometry and IL-10 concentrations by ELISA upon stimulation with the same *M. tuberculosis *antigens and BCG. IL-10 producing cells were not detected in any of the subjects examined by flow cytometry (data not shown) and no secretion of IL-10 was detected in PBMC cultures from control subjects or active TB patients stimulated with PPD, ESAT-6/CFP-10 or BCG (Figure 5B). IL-10 was only detected in supernatants of PBMC stimulated with SEA but no correlation with superantigen dependent IL-10 secretion and TB infection was identified.

## Discussion

Tests based on IFN-γ release after PBMC stimulation with *M. tuberculosis *specific antigens have been recently introduced. These ELISA or ELISPOT tests contribute to the study of T cell responses in TB, and represent an important advance in diagnosis, since they may allow discrimination of BCG-vaccinated from infected individuals [10,15,16,18]. However, a series of limitations to these tests call for a research aimed at ameliorating cytokine release detection [8,13]. In this line, methods to enumerate cytokine-secreting lymphocytes by flow cytometry have been proposed [27,28]. The most promising are based on cytokine detection by intracellular staining, which is possible only after cell fixation and permeabilization with cytokine release blocking substances. *M. tuberculosis *specific cells have low frequencies in PBMC and these procedures do not always guarantee the accumulation of an amount of cytokine high enough to be detected by intracellular antibodies. For this reason some of these methods use co-stimulatory antibodies to increase the cytokine secretion capacity of T cells [27].

In the present work we adapted a commercially available cytokine-secretion method at single living-cell level [22,23] to study T cell responses in *M. tuberculosis *infection. The use of this approach allows the identification and enumeration of living cells actively secreting the cytokines of interest upon specific stimulation, and also their characterization on the basis of membrane expression of selected markers. The frequency of cytokine secreting cells after 72 hours was higher than after 18 hours and sufficient to discriminate with high efficiency *M. tuberculosis*-infected individuals from controls. Due to the characteristics of the method, the increased frequency of secreting cells after 72 hours was not due to an additive effect of cytokines secreted during the three day culture, but it consisted of an increased number of cells effectively secreting cytokines. TEM are terminal cells exerting their functions, such as cytokine secretion, upon encounter with antigen presenting cells (APC). They subsequently are programmed to die [19,29]. Thus, the increased frequency found after a three-day culture is likely to represent the contribution of CEM cells that, upon stimulation, undergo cell division and cytokine secretion [29]. Probably due to the cumulative analysis of both *M. tuberculosis *specific CEM and TEM populations, the specificity and the sensitivity of our test reached high levels. The enumeration of the sole IFN-γ secreting cells upon ESAT-6/CFP-10 stimulation was sufficient to discriminate *M. tuberculosis*-infected individuals from controls. In fact, the contribution of IL-10 and IL-12 secreting cells was irrelevant. Some papers reported levels of IL-10 in TB patients higher than in healthy controls [30]. IL-10 observed in other publications might be dependent on IL-10 release by natural regulatory T cells, that are not stimulated in an antigen specific assay or by using co-stimulating antibodies to increase the cytokine secretion [28]. On the other hand, we observed a trend for IL-2-secreting cells to be detected at frequencies that are higher in individuals with latent TB infection than in those with active disease. The difference was statistically significant when PPD and BCG were used as stimuli, but not significant upon ESAT-6/CFP-10 stimulation. Since the number of epitope specific CEM is relatively small, it is not surprising that responses of increased magnitude are obtained using antigen mixtures such as PPD or the whole mycobacterium (BCG), and not with single antigens such as ESAT-6/CFP-10. These data are likely to reflect the increased number of IL-2 or IL-2/IFN-γ-secreting CEM and the reduced number of IFN-γ-secreting TEM in individuals with latent TB infection in comparison to active TB patients, due to the absence of *M. tuberculosis *replication and, consequently, antigen load [16,21]. However, a larger study is required to confirm these data and to exclude any possible role of environmental mycobacteria.

Our data are in agreement with the recent observation by Millington et al. describing the increase of *M. tuberculosis *specific IL-2 and IL-2/IFN-γ-secreting T cells in the follow-up of active TB treated patients [21]. The emergence of IL-2 secreting cells in patients after antibiotic treatment could be considered as the consequence of the expansion of a new population of cells that can be considered CEM, caused by the reduced mycobacterial antigen load.

Recently, *M. tuberculosis*-specific IL-17 secreting T cells have been described [31,32] and a role of these T helper (Th)17 cells in the pathogenesis of TB hypothesized [33]. Since IL-17 secretion has been associated to chronic inflammations [34,35], we sought to determine its specific secretion in the attempt to identify a surrogate marker of active disease. However, ESAT-6/CFP-10 stimulated PBMC from active TB patients, similarly to control donors, did not secrete IL-17. Only 3 out of 14 patients showed a specific IL-17 secretion upon stimulation with PPD, but these patients showed no clinical signs correlating with the increased IL-17 secretion. Our data confirm that PPD-specific Th17 cells are detected in some active TB patients, but analyses on a larger population would be required to verify whether circulating PPD-specific Th17 cells play any role in disease pathogenesis.

In our assay we noticed the contribution of CD8^+ ^T cells to the cytokine secretion upon *M. tuberculosis *specific stimulation. Although previous papers reported the participation of CD8^+ ^T cells in cytokine secretion [36-38], we were surprised to observe a CD8^+ ^T cell response using PPD and ESAT-6/CFP-10, which are soluble antigens and therefore processed in late-endosomes [39] for presentation to CD4^+ ^T cells. It is possible to speculate that ESAT-6 can be cross-presented [40] by APC after phagocytosis of apoptotic cells, since recent data indicate the capacity of ESAT-6 to induce apoptosis [41]. Moreover, ESAT-6 and other RD1 associated proteins could possibly be directly involved in the endoplasmic reticulum migration from the cytoplasm in a lysteriolysin-like mechanism, as suggested by recent observations [42,43]. Beyond the possible added value for TB diagnosis, the observation of cytokine secretion by CD8^+ ^T cells corroborates the hypothesis of their contribution to the anti-*M. tuberculosis *immune response [44,45] and encourages further studies aimed at correlating CD8^+ ^T cell activation with different clinical stages or prognostic indexes of TB [46].

## Conclusion

We have developed a simple and reliable flow cytometric method to study the antigen specific T cell response in TB. Our assay presents several advantages as compared to other previously described methods based on cytokine release. Moreover, its excellent performance would make it suitable for diagnostic purposes even in field studies [47]. In fact, our preliminary data indicate that detection of IL-2 in IFN-γ positive subjects, after stimulation with *M. tuberculosis *antigens, may discriminate individuals with latent infection from patients affected by active TB. Lastly, since this method allows the contemporaneous evaluation of at least two cytokines it could represent a reference method to track IL-2-secreting T cells and to monitor the efficacy of anti-TB therapy.

## Abbreviations

TB: Tuberculosis; BCG: Bacillus Calmette-Guérin; PPD: purified protein derivative; ESAT-6: early secretory antigenic target-6; CFP-10: culture filtrate protein-10 kDa; IFN: interferon; IL: interleukin; PBMC: peripheral blood mononuclear cells; TST: Tuberculin skin test; RD1: region of difference 1; IGRAs: Interferon gamma release assay; TEM: effector T cells; CEM: central memory T cells; HIV: human immunodeficiency virus; CM: complete medium; SEA: staphylococcal enterotoxin A; FITC: fluorescein isothiocyanate; PE: phycoerythrin; ROC: Receiver operating characteristic; AUC: area under the curve; CI: confidence interval; ELISA: Enzyme Linked ImmunoSorbent Assay; APC: antigen presenting cells.

## Competing interests

The authors declare that they have no competing interests.

## Authors' contributions

VS carried out cytofluorimetric data acquisition and analysis, drafted and revised the manuscript. SC carried out Quantiferon assay. SM and RT were responsible for statistical analysis and contributed to the discussion section. DG was responsible for patient selection and enrolment. DG, MCG and RN were responsible for the conception of the study design and the preparation of the manuscript. All authors read and approved the final manuscript.

## Pre-publication history

The pre-publication history for this paper can be accessed here:

http://www.biomedcentral.com/1471-2334/9/99/prepub
